# *Leptospira* Infections in Cats—What Do We Know?

**DOI:** 10.3390/pathogens15050506

**Published:** 2026-05-08

**Authors:** Bernard Wasiński

**Affiliations:** Department of Bacteriology and Bacterial Animal Diseases, National Veterinary Research Institute, 57 Partyzantów, 24-100 Puławy, Poland; wasinski@piwet.pulawy.pl

**Keywords:** Leptospirosis, *Leptospira*, cat, feline leptospirosis, One Health

## Abstract

The incidence of *Leptospira* spp. infections in cats did not seem to be of major importance until the early 21st century. The relatively rare occurrence of individuals presenting antibodies against *Leptospira* spp. and the almost unheard of clinical cases appeared to suggest that felids are poorly prone to *Leptospira* infections. Considering the close contact of cats with rodents (mice, rats, etc.), which are the main reservoir of leptospires, the above observations may, on the one hand, be surprising, but on the other hand, may reflect species-specific biological or ecological factors influencing susceptibility, although the underlying mechanisms remain poorly understood. The suspicions indicating cats as incidental hosts or asymptomatic carriers of *Leptospira* spp., their proximity to humans, and the “One Health” approach—particularly relevant recently in control of zoonoses—contributed in recent decades to greater research interest in feline leptospiral infections. Recent increasingly frequent data on the occurrence of antileptospiral antibodies in cats, cases of isolation of leptospiral DNA or viable spirochetes from blood or urine samples, and finally cases of clinical disease may support these hypotheses, although the available evidence remains limited and warrants further investigation. This review presents the current data on the incidence and pathogenesis of infections caused by *Leptospira* spp. in cats and their potential epidemiological role, including their possible contribution to environmental contamination and zoonotic transmission.

## 1. Introduction

Leptospirosis is a globally distributed zoonotic disease caused by spirochetes of the genus *Leptospira*. Based on whole-genome sequencing and DNA relatedness, the genus currently comprises over 60 species, traditionally grouped into pathogenic, intermediate, and saprophytic clades [1,2]. Pathogenic leptospires encompass more than 260 serovars, defined by variations in surface-expressed lipopolysaccharide (LPS) antigens and organized into approximately 24–26 serogroups [1,3]. In contrast, non-pathogenic and intermediate species include over 60 additional serovars, some of which have uncertain or variable pathogenic potential [2,4]. Recent genomic studies further refine the taxonomy and highlight substantial genetic diversity among pathogenic and intermediate species.

Leptospirosis can affect virtually all mammalian species, with clinical manifestations ranging from asymptomatic or mild infection to severe, life-threatening multi-organ failure. Nearly every serovar has a primary (maintenance) host that sustains the organism and facilitates its transmission within the environment. Infection with serovars not adapted to a given host is often associated with more severe disease; however, subclinical infections have also been documented in incidental hosts [5].

In addition to mammals, leptospires have been isolated from birds, reptiles, amphibians, and other vertebrates [6,7,8]. These organisms are capable of surviving in the environment, particularly in surface waters, where they may remain infectious for several months [9,10,11,12]. In tropical and subtropical climates, moist environmental conditions represent an important source of human infection. In temperate regions, warm-blooded animals have traditionally been considered the primary reservoir; however, reports of environmentally acquired infections have become increasingly frequent in recent years. Due to the potentially severe and sometimes fatal consequences of human leptospirosis, exposure risk from diverse environmental and biological sources is of major public health concern.

Among companion animals, dogs are most frequently identified as significant reservoirs of *Leptospira*, particularly *L. interrogans* serovars Canicola, Pomona, and Australis. In contrast, clinical leptospirosis in cats has historically been considered rare and was only sporadically reported until recent decades. Notably, as early as the 1910s, Hideyo Noguchi suggested that cats could participate in transmission of spirochetes [13]. The first reports of *Leptospira* isolation from cats date back to the late 1930s, when Mertens (1938) [14] and Esseveld and Collier (1938) [15] described isolates obtained from cats and bats in Batavia (Java). These early feline isolates were mainly associated with serovars related to Javanica, although historical serovar classification has since evolved. Subsequent reports of feline isolates remained sporadic over the following decades.

The first descriptions of naturally occurring leptospiral infection in cats appeared approximately two decades later [16]. Experimental transmission of *Leptospira* serovar Pomona between cats was demonstrated by Ferris and Andrews in the 1960s [17]. Although subsequent decades have seen an increasing number of publications addressing various aspects of feline leptospirosis, current knowledge remains incomplete and fragmented. Major gaps persist regarding the epidemiological role of cats (as carriers, incidental hosts, or sentinels), their susceptibility to different serogroups, the influence of comorbidities and immune status on disease progression, the performance of diagnostic methods, and the effectiveness of treatment and preventive strategies, including vaccination. The actual zoonotic risk posed by cats also remains insufficiently defined.

This review aims to provide a comprehensive synthesis of current knowledge on the prevalence, pathogenesis, clinical presentation, and zoonotic implications of *Leptospira* spp. infection in cats, with particular emphasis on their potential role in environmental contamination and interspecies transmission.

This article was prepared as a narrative review summarizing the current state of knowledge on feline leptospirosis. Literature was identified primarily through searches of PubMed, Scopus, and Web of Science using combinations of keywords including “Leptospira”, “leptospirosis”, “cats”, and “feline”. Both historical and recent publications were included to ensure a broad overview of the topic. Additional relevant studies were identified through reference lists of selected articles. As this is a narrative review, no formal inclusion or exclusion criteria were applied.

## 2. Prevalence

Prevalence data on Leptospira infections in cats vary substantially across continents. The most frequently cited estimates are based on serological studies using the microscopic agglutination test (MAT). A meta-analysis by Andityas et al. (2024) [18] reported the highest seroprevalence in Australia (40.59%), followed by Europe (14.95%), Asia (11.37%), North America (7.21%), and South America (5.06%). Similar results were presented by Ricardo et al. (2023) [19].

These findings provide a general overview of seroprevalence across regions but should be interpreted with caution, as available data—even within single continents—demonstrate marked heterogeneity. For example, in Europe, seroprevalence has been reported as follows: United Kingdom and Czech Republic, 9.2% each [20,21]; Switzerland, 10.3% [22]; Northern Italy, 10.5% [23]; Southern Italy, 15.3% [24]; Estonia, 12.8% [25]; Spain (Andalucia), 13.6% [26] and 4.1% [27]; Germany, 16% [28] and 20.0% [29]; Austria, 18.2% [30]; Serbia, 26.7% [31]; Greece, 33.3% [32]; and France, 48.5% [33].

The observed discrepancies may be due to geographical, climatic, or methodological differences, including cut-off dilutions used in MAT. Due to these uncertainties, no consensus exists regarding the appropriate cut-off in cats. In some studies, the cut-off was set at 1:30 (Agunloye & Nash, 1996) [20], or even 1:20; in others, at 1:40 [33] or 1:50 [29,32], with 1:100 being the most commonly used dilution. For instance, in two German studies, a seroprevalence of 16% was reported at a cut-off dilution of 1:100 [28], and 20.0% at 1:50 [29]. Due to numerous uncertainties and ambiguities, no consensus has yet been reached regarding the appropriate cut-off dilution in serological testing of cats using MAT.

According to the European consensus statement, the most frequently identified Leptospira serogroups in cats in Europe are Icterohaemorrhagiae, Canicola, Grippotyphosa, Pomona, Sejroe, Ballum, Autumnalis, and Bratislava [34]. In the United States, the most commonly detected serogroups in felines are Australis, Autumnalis, Grippotyphosa, and Pomona [27].

Published studies on the detection of *Leptospira* DNA in feline urine samples report a wide range of prevalence rates, from 0.8% in Thailand [35] to 67.4% in Taiwan [36]. Importantly, PCR results should be interpreted cautiously, as detection of leptospiral DNA may reflect transient bacteremia or temporary shedding rather than persistent infection. In Hong Kong, urinary shedding of *Leptospira* DNA was detected in 12 out of 268 (4.48%) community cats [37]. Investigators from Malaysia detected *Leptospira* DNA by PCR in 4 out of 82 cats (4.9%) and additionally isolated *Leptospira*, identified as serovar Bataviae, from the urine or from both urine and kidneys of four other cats [38]. Only one of the culture-positive cats (with leptospires detected in both urine and kidneys) demonstrated antibodies in MAT at a titer of 1:100. *Leptospira* DNA was not detected in the urine samples of these culture-positive cats. However, all four cats with *Leptospira* DNA detected in urine samples also showed positive MAT results.

Data from several European countries also demonstrate considerable variation. In southern Italy, 9% of feline urine samples and 3% of blood samples tested positive for *Leptospira* by PCR [24]. In Germany [39], Croatia [40], and Spain [27], the prevalence of *Leptospira* DNA in feline urine samples was reported as 3.3%, 1.85%, and 1.72%, respectively.

On the American continent, results similar to those obtained in Germany were reported in Canada (3.4%) [41]. In the United States (Colorado), 11.7% of feline urine samples tested positive by PCR [42] (conference abstract). In South America, a study conducted in Chile analyzing 231 urine samples from outdoor cats in both rural and urban areas revealed the presence of *Leptospira* DNA in 7.8% of the animals using the qPCR method and in 13% using the immunomagnetic separation (IMS)-coupled qPCR (IMS-qPCR) technique [43]. Interestingly, three of the investigated cats were culture-positive, and four additional cats tested positive using immunomagnetic separation coupled with culture (IMS-coupled culture). Only one of the cats that tested positive in IMS-coupled culture was also positive in both qPCR and IMS-qPCR. The remaining six cats were negative in both PCR-based methods [43].

Data from the Australian region are sparse. However, an investigation of 59 feral cats from Christmas Island detected leptospiral DNA in the kidneys of 25 animals (42.4%) [44]. In contrast, the same study conducted in southwestern Western Australia and on Dirk Hartog Island did not detect pathogenic leptospires in any of the examined cats.

As may be observed in at least some of the reports mentioned above, the results of investigations using PCR are relatively rarely confirmed by the isolation of *Leptospira* or even by serological test results (MAT). This may reflect not only the limitations inherent to individual diagnostic methods (e.g., *Leptospira* isolation, which is time-consuming, highly dependent on culture conditions, and characterized by low sensitivity), but also factors related to the pathogenesis of the infection or the stage of the disease. A positive PCR result, in the absence of serological or culture confirmation, may indicate transient leptospiremia, chronic infection, or residual DNA following bacterial clearance. It should also be emphasized that the specificity of PCR-based assays may vary depending on the target gene and primer design. While commonly used targets such as lipL32 are generally considered specific for pathogenic *Leptospira* spp., differences in assay design and laboratory protocols across studies may influence both sensitivity and specificity. In rare cases, non-specific amplification or detection of closely related genetic material cannot be entirely excluded, particularly in the absence of sequencing confirmation. These methodological differences further complicate comparisons between studies and the interpretation of PCR-positive results. Extracellular DNA and potential laboratory contaminants may also contribute to false-positive PCR results, thereby complicating interpretation.

In contrast, a positive MAT result accompanied by negative culture and PCR findings may indicate a past infection without active bacterial shedding or even the absence of detectable bacterial DNA. Subclinically infected cats may fail to mount a detectable humoral immune response, and antibody titres may remain low or fall below the detection threshold. Additionally, certain *Leptospira* serovars or strains may not induce a sufficiently strong immune response to be reliably detected serologically. On the other hand, the isolation of *Leptospira*, despite remaining the gold standard for laboratory diagnosis, has limited applicability in routine practice due to its low sensitivity, the prolonged incubation period often required for bacterial growth (frequently several weeks), and its susceptibility to contamination and inhibitory substances.

The results presented above, although relatively numerous, are often heterogeneous and only infrequently include bacteriological, molecular, and serological findings within the same study. Variation in results, even within individual diagnostic method categories, as well as inconsistencies—or apparent inconsistencies—between serological, bacteriological, and molecular data, complicate interpretation and contribute to the fact that our understanding of *Leptospira* infection prevalence in cats still remains limited and somewhat general.

## 3. Pathogenesis

Current knowledge regarding the pathogenesis of *Leptospira* infections in felids is considerably less detailed than that available for other mammalian species, such as dogs. Nonetheless, recent evidence allows outlining the general course of these infections in cats. Overall, the infection course appears to resemble that observed in other mammals.

Leptospires enter the host through damaged skin or mucous membranes, and transmission occurs via exposure to contaminated environments or ingestion of infected animals [45,46]. In the host organism, spirochetes spread via the bloodstream to organs such as the kidneys, liver, lungs, and other tissues. Leptospiraemia in cats is likely subclinical or short-lived, potentially lasting 4–7 days, though exact duration is undetermined [47,48,49]. This uncertainty may reflect subclinical or asymptomatic leptospiraemia, rapid antibody response, or low circulating spirochete concentrations [47].

In humans and many mammals, endothelial damage caused by leptospires in small vessels can lead to localized ischemia, inflammation, and organ dysfunction. Leptospires penetrate renal epithelial or hepatic parenchymal cells, evading immune detection and establishing a permissive niche for replication.

Direct evidence of organ colonization in cats remains limited. Most studies confirm renal and urinary tract involvement, with occasional liver and other tissues affected [17,24,38,41,50,51]. Spirochetes can persist in renal epithelial cells, leading to nephritis of varying severity. Some studies suggest a possible link between *Leptospira* infection and chronic kidney disease (CKD) in cats; however, this has not been unequivocally confirmed.

Leptospiral toxins may damage hepatocytes, leading to necrosis, occasional neutrophilic hepatitis, and, if persistent, increased fibrosis risk due to impaired perfusion [52,53]. Direct evidence of liver colonization in cats is scarce and mainly based on bacterial isolation [16,17,50]. Biochemical parameters, including mild to moderate alterations in ALT, AST, ALP, and bilirubin levels, may reflect functional hepatic changes [24,45]. Hyperbilirubinemia occurs less frequently in cats than in dogs, and elevated ALT may indicate acute or subclinical hepatocellular injury.

Leptospiral Pulmonary Hemorrhage Syndrome (LPHS), common in humans and dogs, has not been reported in cats. This may relate to the infection route, typically through ingestion or aspiration of contaminated water while swimming.

Beyond kidneys and liver, leptospires have been isolated from thoracic fluid and aqueous humor, and detected in brain tissue by silver impregnation techniques [51].

Colonization of renal tubular epithelial cells marks the transition to a potential carrier (shedding) state, although the duration and epidemiological relevance of this state in cats remain uncertain. Shophet and Marshall (1980) [54] reported experimentally infected cats excreting up to 10^4^ leptospires/mL urine. Leptospiruria generally begins around two weeks post-infection and may persist up to 60–80 days [47,48,54]. Some shedding cats may test negative in MAT, potentially due to intracellular localization of spirochetes shielding them from circulating antibodies [19,38].

Effective transmission between cats may occur when shedding individuals encounter susceptible hosts, with risk influenced by bacterial load, environmental persistence, host immunity, and concurrent infections (e.g., feline panleukopenia virus) [23].

## 4. Clinical Signs

Cats seroconvert after leptospiral infection but are less prone to develop overt clinical symptoms [51,55,56]. The influence of age, sex, breed, lifestyle, and contact with reservoir hosts on clinical manifestations remains unclear. Few studies report clinical feline leptospirosis, and early research suggested that infections were often inapparent [20,48,49,57]. This led to hypothesis of natural resistance or underdiagnosis. However, more recent evidence confirms that natural infections do occur [58,59].

Reports of naturally occurring leptospiral infections in cats remain rare worldwide [49,51,55,56,58,59,60]. Initial studies failed to establish a link between infection and interstitial nephritis [61], but subsequent research has shown a clearer association [49,51,55,56,60]. Clinical signs, when present, may include fever, polyuria/polydipsia, hematuria, diarrhea, vomiting, ascites, jaundice, uveitis, lethargy, anorexia, weight loss, and poor body condition [16,20,58,59,60,62]. Additionally, leptospiruria and/or detection of *Leptospira* DNA have been documented [36]. Fatality rates can reach 50%, with spontaneous death or euthanasia reported.

Hematologic and serum biochemical abnormalities in cats with leptospiral infection are generally nonspecific and milder than those typically observed in dogs. The most common hematologic findings include leukocytosis with neutrophilia, and less frequently, mild normocytic normochromic anemia or thrombocytopenia [55,56,58].

Laboratory abnormalities primarily reflect renal or hepatic involvement. Azotemia, associated with acute kidney injury, is regularly observed in clinically affected cats, often alongside alterations in electrolyte balance, including hypo- or hyperkalemia, depending on the stage of tubular dysfunction. Hepatic involvement is usually characterized by mild to moderate increases in ALT, AST, and ALP, whereas hyperbilirubinemia appears less frequent in cats than in dogs [24,63].

Urinalysis commonly reveals proteinuria, isosthenuria, and evidence of tubular damage such as glucosuria without hyperglycemia or granular casts. Leptospiruria can be detected by PCR, although its intensity and duration vary widely among infected cats.

Despite available case reports and small series, understanding of hematologic and biochemical changes in cats with leptospiral infection remains limited. Current data are largely descriptive, derived from isolated cases without standardized diagnostics, and do not clarify the prevalence, severity, or progression of laboratory abnormalities. The influence of bacterial load, serovar, carrier status, and concurrent diseases on these changes remains unknown. Prospective studies with larger sample sizes and standardized monitoring are needed to define typical laboratory profiles and distinguish primary leptospiral effects from comorbidities.

## 5. Treatment

Clinical management of leptospirosis in cats largely resembles that used in dogs, including antibiotic therapy and supportive care aimed at managing renal involvement [27,62,63,64,65,66].

Antibacterial therapy is tailored according to the clinical condition of the animal, as well as the stage and course of the infection.

In the early stage of infection, which may occasionally present with acute clinical signs, antibiotic administration aims to rapidly inhibit leptospiral multiplication and to limit potentially life-threatening complications, such as vomiting, uraemia, and hepatic dysfunction. At this stage, parenteral (mainly intravenous) administration of penicillin derivatives—primarily ampicillin or amoxicillin—is recommended [27,64,65,66]. These agents effectively eliminate leptospires from the bloodstream and reduce the risk of transmission but do not eradicate the organisms from the kidneys, and therefore do not prevent long-term renal colonization.

During the second stage of therapy, oral doxycycline is considered the drug of choice. Parenteral administration of doxycycline in cats is not recommended due to adverse effects, including vomiting and reported cases of shock following intravenous injection, as well as abscess formation after subcutaneous administration. When given orally, doxycycline monohydrate is the least irritating formulation for the feline esophagus, and a liquid suspension is preferred over tablets or capsules. Initiation of oral doxycycline therapy is recommended only after resolution of vomiting and normalization of hepatic enzyme activity [65].

In animals without clinical signs or with only mild manifestations, doxycycline may be used instead of penicillin derivatives even during the early stage of infection. Oral doxycycline can also be administered to asymptomatic cats in which leptospiral shedding has been detected; in such cases, treatment is recommended for a duration of three weeks.

In addition to antibacterial therapy, supportive care is particularly important during the early stage of infection. This includes correction of fluid and electrolyte imbalances (intravenous fluid therapy), administration of antiemetic drugs, analgesic therapy, and medications protecting the gastric mucosa [27,65].

## 6. Prevention and Control

No licensed vaccines are available for cats; therefore, preventive strategies rely on reducing exposure and risk factors. Experimental studies show that cats can develop antibodies after exposure to canine vaccines, but responses are variable and short-lived [62,67]. Available leptospirosis vaccines for dogs, as in humans, provide protection primarily against specific serovars. Duration of immunity varies by formulation, and vaccines do not completely prevent renal colonization or shedding. These data provide context for discussing preventive strategies in cats.

Notably, the immune systems of cats and dogs differ, which has implications for vaccine design and scheduling, particularly in young animals [68]. Cats also appear more prone to post-vaccination reactions and require greater caution regarding adjuvant selection, supporting the need for tailored formulations [69].

In developing vaccines for cats, critical considerations include immune responses to antigens and adjuvants, safety and minimization of adverse events, experimental determination of duration of immunity, optimization of dosing and booster schedules, and clinical evaluation of both disease prevention and potential effects on bacterial shedding. Current research directions include epidemiological studies on the role of cats as reservoirs, improved understanding of feline immunological responses to *Leptospira*, and translational vaccine research exploring novel formulations such as recombinant or subunit vaccines with engineered adjuvants. However, to date, no clinical data demonstrate vaccine efficacy in cats.

Key gaps in our understanding of prevention in cats remain the immunological correlates of protection, the duration and strength of humoral and cellular responses, the potential impact of vaccination on subclinical infection or shedding, and the contribution of cats to environmental contamination. Addressing these gaps is essential for informing the development of feline-specific vaccines or other targeted preventive strategies.

## 7. Epidemiological and Zoonotic Aspects—One Health Perspective

Leptospirosis in cats is predominantly subclinical, with most infected individuals showing no overt clinical disease despite serological evidence of exposure. Systematic reviews and meta-analyses report a global seroprevalence of approximately 10–12% in domestic cats and detection of pathogenic *Leptospira* DNA or organisms in the urine of a subset of cats, indicating that these animals can be exposed to infection and intermittently shed leptospires in their environment [62].

Although cats can shed pathogenic *Leptospira* in urine, the epidemiological significance of cats as reservoirs is limited and incompletely understood [43].

Available data do not provide definitive, epidemiologically confirmed cases in which transmission has been traced from cats to humans or other animal species with molecular linkage of identical strains. However, the presence of leptospiral DNA or isolates in feline urine confirms that cats may contribute to environmental contamination, which may indirectly contribute to transmission cycles in multi-host ecosystems [62].

Compared with established maintenance hosts such as rodents, livestock, and domestic dogs—which are recognized reservoirs for specific serovars and have clearer epidemiological links to human infection—the role of cats in the maintenance and transmission of leptospiral infection is generally considered to be of lower epidemiological significance [62].

Nonetheless, outdoor cats and those with access to wildlife or contaminated environments show higher probabilities of seropositivity and urinary shedding, supporting the hypothesis that these animals can serve as environmental sentinels for circulating *Leptospira* in a given ecosystem [19]. Current preventive strategies for feline leptospirosis focus primarily on non-specific measures (e.g., reducing outdoor exposure, limiting contact with potentially contaminated water and reservoir hosts), as specific prophylaxis through vaccination or targeted interventions remains underdeveloped and not routinely recommended in clinical practice [62]. The potential impact of antimicrobial therapy or hypothetical vaccination on transmission dynamics, environmental contamination, or public health outcomes has yet to be established.

From a One Health perspective, integrated control strategies should also consider environmental and reservoir-related factors, particularly the role of rodents as primary maintenance hosts of *Leptospira* spp. Measures aimed at reducing environmental contamination, including rodent population management in urban and peri-urban settings, as well as improving sanitation and limiting access of cats to high-risk environments, may contribute to lowering exposure risk. In addition, community-level awareness and surveillance programs may support early detection of infection sources and enhance prevention efforts, although their effectiveness in the context of feline leptospirosis remains to be clearly established.

Given these knowledge gaps, future research should adopt a One Health perspective, integrating epidemiological surveillance, molecular typing, and environmental studies to clarify (1) the contribution of cats to *Leptospira* circulation in various ecological settings, (2) their potential role in interspecies transmission, and (3) the evidence base for effective preventive strategies aimed at mitigating zoonotic risk.

## Data Availability

No new data were created or analyzed in this study.
